# Awakening effects of blue-enriched morning light exposure on university students’ physiological and subjective responses

**DOI:** 10.1038/s41598-018-36791-5

**Published:** 2019-01-23

**Authors:** Kyungah Choi, Cheong Shin, Taesu Kim, Hyun Jung Chung, Hyeon-Jeong Suk

**Affiliations:** 1 Department of Industrial Design, Korea Advanced Institute of Science and Technology (KAIST), Daejeon, 34141 Republic of Korea; 2 Graduate School of Nanoscience and Technology, Korea Advanced Institute of Science and Technology (KAIST), Daejeon, 34141 Republic of Korea

## Abstract

We investigated physiological and subjective responses to morning light exposure of commercially available LED lighting with different correlated colour temperatures to predict how LED-based smart lighting employed in future learning environments will impact students. The classical markers of the circadian system (melatonin and cortisol), as well as the subjective perception of sleepiness, mood, and visual comfort, were compared. Fifteen university students underwent an hour of morning light exposure to both warm (3,500 K) and blue-enriched (6,500 K) white lights at recommended illuminance levels for classrooms and lecture halls (500 lux). The decline of melatonin levels was significantly greater after the exposure to blue-enriched white light. Exposure to blue-enriched white light significantly improved subjective perception of alertness, mood, and visual comfort. With regard to cortisol, we did not find a significant difference in the cortisol decrement between the two light conditions. Our findings suggest that the sensitivity of physiological and subjective responses to white LED light is blue-shifted. These findings, extending the already known effects of short-wavelength light on human physiology, reveal interesting practical implications. Blue-enriched LED light seems to be a simple yet effective potential countermeasure for morning drowsiness and dozing off in class, particularly in schools with insufficient daylight.

## Introduction

In 2002, it was established that a novel, third type photoreceptor regulates a large variety of biological and behavioural processes^1,2^, including melatonin^3–6^ and cortisol^5–8^ secretion, alertness^3,5,8,9^, electroencephalogram (EEG)^8,9^, and functional magnetic resonance imaging (fMRI)^10,11^. These photoreceptors, called intrinsically photosensitive retinal ganglion cells (ipRGCs), have neural connections to the suprachiasmatic nuclei (SCN) of the hypothalamus, which serves as the circadian clock in an organism. The maximum sensitivity of the ipRGCs lies in the short-wavelength blue region (peak sensitivity at about 460 nm) of the spectrum^12,13^, which has led to the generation of a term called “blue light.” Such advances in research, coinciding with the introduction of smart light technology that can be integrated into the Internet of Things (IoT) platform and tuned to emit any colour^14^, have brought about a wider range of possibilities to lighting practice with the promise of new design paradigms.

Although it is acknowledged that students spend approximately 85% of their time indoors^15^, surprisingly little effort has been made to apply the research findings to learning environments. From the educational standpoint, the choice of light to be used has been based mainly on its effects on the cognitive performance of students. The literature suggests that blue-enriched white light leads to higher alertness and better performance during intensive academic activities, whereas warm white light is associated with relaxing ambience and improved communication^15–18^. However, for optimally applying lighting, it is increasingly important to understand the physiological influences light may exert in learning environments. The effective design of school buildings provides daylight as an effective light source for regulating the circadian system^19^. However, in many cases, there is not enough daylight in schools, especially in northern latitudes in the winter^20^. The lack of sufficient daylight clashes with early morning school schedules, resulting in morning drowsiness and dozing off in class. Where the benefits of daylight are not sufficiently available, dynamic electric light can be advantageous.

Although initial studies clearly showed the effectiveness of blue monochromatic, fully saturated lights for stimulating physiological responses^3–11^, the relative effectiveness of polychromatic, white light sources is less well understood. The ability to understand the impacts of white light sources is especially important because indoor environments are exclusively illuminated with these light sources. Recently, white light sources have become available in a wide range of correlated colour temperatures (CCTs), a measure of light source colour appearance along a warm–cool dimension. Light with a high CCT appears bluish white, containing more energy in the short wavelength region of the spectrum compared to light with a low CCT. Because lights with different CCTs are composed of different wavelengths, they might also exert different physiological impacts on humans.

Much of our knowledge about the impacts of white light sources comes from studies during night-time, when people are normally asleep. The night-time studies using fluorescent lamps unequivocally reported the superiority of higher CCT light in stimulating the circadian system^21–25^, whereas only a few studies have focused on the impact of morning light exposure. Jung *et al*.^26^ showed the acute effect of bright morning light exposure on cortisol levels, while Rüger *et al*.^27^ failed to find such an effect. Aside from the circadian phase of cortisol, Sato *et al*.^28^ observed significant melatonin suppression under a higher CCT fluorescent lamp of 2,500 lux in the morning. Gabel *et al*.^5^ observed the effects of artificial dawn light on cognitive performance, subjective perception of well-being and mood, and melatonin and cortisol levels in comparison with blue monochromatic light. To further highlight the potential of using morning light, some researchers expanded their scope of research to investigate whether morning light exposure influences melatonin profiles in the evening^29–31^.

The inconsistent findings of earlier studies indicate that the impacts of light highly depend on the research design, including the intensity, timing, and duration of the light exposure. Recently, the use of light-emitting diode (LED) has continued to increase owing to the low energy consumption and governmental regulations^32,33^. The growth is powered by the IoT integrated into LED-based luminaires, which is also called smart lighting^34^. The light sources covered by earlier studies were limited to incandescent and fluorescent bulbs, leaving open the question of how LED-based smart lighting can be utilized in learning environments to stimulate the circadian system. Moreover, the light conditions employed in earlier studies were not aligned with the standardized recommendations for educational buildings^35,36^, making it difficult to relate research findings to applications in learning environments. To address this question, we investigated the change in physiological and subjective responses during the morning to commercially available LED lighting with different CCTs. We hypothesized that melatonin and cortisol, two marker rhythms of the circadian system, as well as the subjective perception of sleepiness, mood, and visual comfort, would be differentially affected by the morning administration of 3,500 K and 6,500 K LED lightings, following the standards for learning environments.

## Results

Fifteen healthy young adults (eight men and seven women; mean ± s.e.m. age of 23.53 ± 0.87) completed the experiments. The study consisted of two light conditions: 3,500 K warm white light (WL) and 6,500 K blue-enriched white light (BL). All participants were exposed to both conditions, but the order was randomized. Saliva samples were taken before and after the light exposure for assessment of melatonin and cortisol concentrations. Subjective sleepiness was rated using the Karolinska Sleepiness Scale (KSS). Participants rated their subjective mood and visual comfort on 100 mm visual analogue scales (VASs). More detailed methods are described in *Methods*.

### Salivary Melatonin

**Figure 1:**
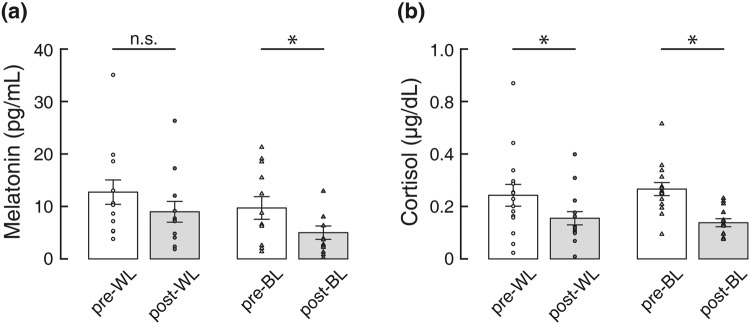
Changes in (**a**) salivary melatonin (n = 11) and (**b**) cortisol levels (n = 15) before and after the light exposure. The data for WL and BL conditions are plotted with circles and triangles, respectively. An asterisk indicates significance at *p* < 0.05. The means ± s.e.m. of experimental results are shown.

Figure 1 shows salivary melatonin concentrations before and after light exposure. The Wilcoxon signed-rank test showed that melatonin levels were significantly lower only following BL exposure (*Z* = −2.93, *p* = 0.003). There was no significant difference between melatonin levels recorded before and after WL exposure (*Z* = −1.69, *p* = 0.091). Raw data were then converted to the percentage change as described by [(post hormone–pre hormone)/pre hormone] × 100. Comparison of percentage of melatonin changes indicated that salivary melatonin concentration decreased 20.17% and 53.18% after WL and BL exposure, respectively (Table 1). A paired samples t-test indicated that the percentage of melatonin changes differed statistically significantly between the two lightings (*t*(10) = 2.78, *p* = 0.019, Cohen’s *d* = 0.84).

**Table 1:** Results of various physiological and subjective measurements. An asterisk indicates significance at *p* < 0.05. The means (s.e.m.) of experimental results are shown.

Measure	WL	BL
Melatonin change (%)*	–20.17 (12.68)	–53.18 (9.24)
Cortisol change (%)	–13.36 (20.88)	–43.00 (6.12)
Subjective sleepiness*	6.47 (0.49)	5.07 (0.45)
Subjective mood*	48.20 (4.33)	57.13 (5.45)
Subjective visual comfort*	54.83 (5.71)	61.60 (5.77)

### Salivary Cortisol

Comparisons of salivary cortisol levels are shown in Fig. 1. The Wilcoxon signed-rank test yielded a significant decrease in cortisol levels after both WL (*Z* = −2.44, *p* = 0.015) and BL (*Z* = −3.41, *p* = 0.001) exposure. Comparison of percentage of cortisol changes indicated that salivary cortisol concentration decreased 13.36% and 43.00% after WL and BL exposure, respectively (Table 1). However, such a decrease in cortisol levels did not significantly differ between light conditions (*Z* = −0.97, *p* = 0.334).

### Subjective Perception of Sleepiness, Mood, and Visual Comfort

The ratings of subjective sleepiness, mood, and visual comfort were calculated for each light condition (Table 1). There was a statistically significant effect of light condition on the subjective perception of sleepiness (*t*(14) = 2.72, *p* = 0.017, Cohen’s *d* = 0.70). Participants felt less sleepy after an hourly exposure to BL when compared to the exposure to WL. Mood rating was significantly higher for BL compared to WL (*t*(14) = 2.24, *p* = 0.042, Cohen’s *d* = 0.58). There was also a significant effect of light condition on subjective visual comfort (*t*(14) = 2.78, *p* = 0.015, Cohen’s *d* = 0.72). BL was subjectively perceived as more comfortable compared to WL.

### Light Measurements

**Figure 2:**
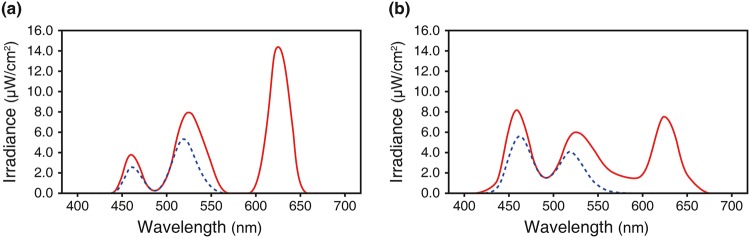
The spectral power distribution (solid line) with melanopic weighted power (dashed line) for the two light conditions: (**a**) warm white light and (**b**) blue-enriched white light.

Table 2 displays the measurements of two light conditions according to the basic parameters used in architectural lighting to quantify light colour and intensity. The measured spectral power distributions of the two light conditions are shown in Fig. 2. As can be seen, BL produced a considerably higher output between the 440 and 480 nm short wavelength region of the spectrum, with a peak at 460 nm, compared with WL, with a peak at 625 nm. Table 3 presents the effective irradiance experienced by each of the rods, melanopsin, and S, M, and L cones, namely rhodopic, melanopic, cyanopic, chloropic, and erythropic illuminance, in comparison with photopic illuminance (see *Methods*). Two stimuli were matched in terms of standard measure of brightness (516.14 vs. 518.38 photopic lux) but differed in their excitation of melanopsin (410.33 vs. 586.86 melanopic lux) and S cones (176.58 vs. 525.64 cyanopic lux). BL provided 177 more melanopic lux than WL (43% more), and there was a difference of 349 cyanopic lux (around threefold).

**Table 2:** Measurements of two light conditions in terms of the visual properties of light.

Measure	WL	BL
CIE 1931 coordinates (*x*, *y*)	(0.4093, 0.4134)	(0.3126, 0.3214)
CCT (K)	3590	6575
Photopic illuminance (lux)	516.14	518.38
Irradiance (µW/cm^2^)	164.08	179.25
Photon flux (photons/cm^2^/s)	4.71 × 10^14^	4.88 × 10^14^

**Table 3:** Comparison of two light conditions for the photopic illuminance and effective irradiance experienced by each of the rods, melanopsin, and S, M, and L cones.

Measure	Spectral Sensitivity	WL	BL
Photopic lux	Visibility	516.14	518.38
Rhodopic lux	Rod	463.95	581.28
Melanopic lux	Melanopsin	410.33	586.86
Cyanopic lux	S Cone	176.58	525.64
Chloropic lux	M cone	455.94	527.47
Erythropic lux	L cone	506.89	510.52

## Discussion

We investigated whether an hour of morning light exposure with different CCTs would differently affect university students’ physiological and subjective responses. In particular, we aimed to observe how the known effects of monochromatic blue light on the human circadian system translate to future learning environments illuminated by white LED lighting. The classical markers of the circadian system (melatonin and cortisol) as well as the subjective perception of sleepiness, mood, and visual comfort were compared.

We observed that the decline in melatonin secretion was greater with exposure to blue-enriched white light in comparison to warm white light. Our data are in line with previous observations indicating that non-visual responses are highly sensitive to short wavelength light^3–5,21–24,28–30^. While the majority of previous studies focused on the effects of night-time light exposure on non-visual responses^3,4,21–24^, this experiment demonstrated such an effect in the morning with commercially available white LED light. Interestingly, the amount of melatonin secreted was quite high, which might be due to the fact that the experiments were carried out during the winter season (December to January). According to previous studies, melatonin levels are higher during the winter, and melatonin circadian rhythm also displays a phase shift towards the morning hours in the winter^37,38^. Hence, the magnitude of our data should be viewed with caution.

With regard to the cortisol, we did not find a significant difference in the cortisol decrement between the warm and blue-enriched white light exposure. This lack of effects might be due to our timing of the light exposure, which was most likely applied too late to induce any cortisol response. The effects of blue light on cortisol have only been reported previously when applied in the early morning hours, close to wake up time^5,7^. Furthermore, although we observed a decrease of cortisol levels, light exposure during the morning has been reported to increase^5,7^ or have little effect^6^ on cortisol levels in humans. Our results are similar to previous observations reporting that morning cortisol levels decreased after light exposure when the participants had been awake for a few hours^26^. Hence, future works to understand the effects of light on the cortisol secretion will need to address such experimental confounds.

Lastly, we found that morning exposure to blue-enriched white light significantly improved subjective perception of alertness and positively affected mood and visual comfort. Until recently, it has been hypothesized that melatonin is involved in these effects via SCN-dependent mechanisms^39^. However, other possibilities should also be considered. Besides the SCN, candidate retinal projections for these effects might possibly be the amygdala, a core component of the emotional brain that receives direct projections from ipRGC^11^. Thus, it would be valuable to conduct an fMRI study to investigate the neuronal correlates of subjective responses to white light with different CCTs.

The spectral composition may explain why the magnitude of physiological and subjective responses shifted towards one particular light between two light conditions that have same photopic lux. In normal architectural lighting design, photopic illuminance has been nearly the one and only criterion used^40,41^. However, the study shows that photopic illuminance is an inappropriate measure for estimation of the impact of light on a wide array of non-visual effects. Aside from the conventional measures, a new light-measurement strategy proposed by a group of researchers^42^ should be taken into consideration by interior designers, architects, and engineers to fully evaluate the relative merits of different lightings based on their visual and non-visual impacts.

There are several features of the research design that may have affected the quality of the findings. A key limitation is the lack of a control condition in dim light, resulting in a partial picture of the effects observed in this study. Further research should be performed using a control group to fully elucidate the effects of morning light exposure. The next concern is the time of assessments. In the research presented, the experiments were conducted from 9:00 to 11:00, considering participants’ wake up time and the time that it took them to get to the laboratory. However, there is a need for research earlier in the morning to be applicable to the earliest classes when students would be most affected by drowsiness. Moreover, although Sato *et al*.^28^ observed significant melatonin suppression under a higher CCT fluorescent lamp immediately after wake up, no studies have reported the acute melatonin suppression effects around 10:00 to 11:00. Earlier studies were focused on the impacts of mid- to late-morning light administration on the melatonin profiles at night^29–31^. In sum, the data should be viewed with caution because our results may have been diminished or enhanced by the influence of multiple factors. The study can also not exclude the possibility that the participants were affected by the withdrawal from caffeine on the study day. Therefore, a better understanding will demand setting up long-term quasi-experimental field studies. It would also be valuable to investigate inter-individual differences in response to light, including different age groups^25^ and chronotypes^43^. Another avenue for further study would be to investigate if the suggested effects of lighting CCTs differ among different light sources. In particular, organic light-emitting diodes (OLEDs) will likely generate new applications owing to the unique properties of their large-area diffused light with adjustable colour^44^. Hence, further research is needed for universal applications of the research findings.

Nevertheless, the findings from this study reveal some interesting practical implications. Blue-enriched LED light exposure might be an effective potential countermeasure for morning drowsiness and dozing off in class, particularly in schools with insufficient daylight. From the educational standpoint, however, warm white light has been reported to provide a relaxing environment and support communication^15–18^. Therefore, application of blue-enriched white light requires careful consideration and must be incorporated appropriately according to learning activities. Otherwise, an auto-dimming feature could be suggested, in which the warm white light gradually changes to blue-enriched white light after its prolonged use during the morning.

There is still much that we need to know before bringing the dynamics of light into learning environments, but not much progress can be made if we continue to apply conventional lighting. When acknowledging all of the physiological and subjective effects that are unique to light, we should go one step further and elaborate upon the potential that lighting can have to function as a platform to support students’ circadian systems. Although further research is necessary, this study is expected to provide the basis for major changes in future lighting strategies and thereby shed light on better learning environments.

## Methods

### Participants

Participants were recruited from a campus population through Internet advertisement and flyers. The screening procedure began with an email interview involving instruction about the experimental procedure and the possible side effects of the experiment. All participants gave written informed consent and were paid for their participation. The study protocol, screening questionnaires, and consent form were approved by the Institutional Review Board (Korea Advanced Institute of Science and Technology, Republic of Korea). All experiments were performed in accordance with relevant guidelines and regulations of the committee.

All applicants completed questionnaires about their sleep quality, life habits, and health state. These questionnaires comprised a consent form, a Pittsburgh Sleep Quality Index (PSQI)^45^, and Morningness–Eveningness Questionnaire (MEQ)^46^. Candidates with a PSQI score of five or greater, as well as definite morning and definite evening types, were excluded. Exclusion criteria also included colour deficiency, smoking, medication or drug consumption, history of chronic health problems, psychiatric disturbance, sleep disorder, and shift work and transmeridian flights taken during the month prior to the study. The average habitual sleep time of participants was 24:30 ± 0.30 hours (mean ± s.e.m.), and average habitual wake up time was 8:08 ± 0.29 hours (mean ± s.e.m.). All participants were classified as low and moderate caffeine users (less than 200 mg per day)^47^.

Participants were grouped into three teams, with five participants on each team. Beginning a week prior to the experiment onset, participants were instructed to keep a regular sleep−wake schedule (sleep onset between 23:00 and 24:00 and wake between 7:00 and 8:00). The sleep−wake schedule was confirmed by a wearable sleep tracker (Xiaomi Inc., China) and self-reported sleep logs. They were asked to refrain from excessive napping as well as heavy alcohol and caffeine intake for the entire experimental period, beginning a week prior to the experiment onset, to level out the impact of these behaviours on the variables reported here. Moreover, they were instructed to abstain from napping as well as alcohol and caffeine consumption at all the day before the experiment. A total of fifteen young adults (eight men and seven women) who fulfilled all inclusion criteria were recruited. The average age of the participants was 23.53 years, with a s.e.m. of 0.87 years.

### Study Design

The data presented in this paper come from a larger study involving evening study (see Supplementary Data for more details). The experiments were carried out during the winter season (December to January) in Daejeon, Republic of Korea. The experiments started at 9:00, and the subjects were exposed to one of the two lighting treatments in a random order. On their way to the laboratory, participants were required to wear dark goggles to avoid light exposure before the study sessions. Each experiment was separated by a 3-day intervening period. Although subjects conducted a normal life during these intervals, they were instructed to maintain a regular sleep−wake schedule.

Each session lasted for two hours. Participants underwent an episode of one hour under dim light conditions (<10 lux). Subsequently, light exposure was initiated for the next hour. During the experiment, they were instructed to sit on a chair and watch educational documentary films. The documentary films were projected on the wall (<10 lux) because bright light from the display has the possibility of affecting melatonin and cortisol secretion. They were instructed to remain awake during the experiment. Outcome measures included the amount of melatonin and cortisol secretion, as well as the self-reported ratings of sleepiness, mood, and visual comfort. The ambient conditions of the experimental chamber were maintained for human comfort: ambient temperature = 21.2 ± 1 °C; ambient humidity = 35 ± 2%; and ambient noise = 64.2 ± 10 dB(A).

### Light Conditions

The study was conducted in a room equipped with an LED luminous ceiling. The room was refurbished to appear like a classroom and was neutral in colour. A uniform illumination environment was constructed in the room, which illuminated the entire space, ensuring that the participants fully adapted to the surrounding environment. The light conditions were manipulated using a specification widely used in industry to quantify light sources. In most architectural lighting design, illuminance is often the only criterion used to design a lighting solution^40,41^. The illuminance was adjusted to 500 lux, which is recommended for classrooms and lecture halls^35,36^. As for the lighting CCT, 3,500 K and 6,500 K CCTs were selected, referring to the lower and upper boundaries of typical electric lights commercially available in the market. The light measurements were taken using a spectrometer (IM‐1000, Topcon) and a colourimeter (CL‐200 A, Konica Minolta). In order to provide a description of light as experienced by the circadian, neuroendocrine, and neurobehavioral systems, five distinct biological representations of irradiance were calculated. The irradiance output in the range of 380–780 nm at 5-nm intervals was used to calculate the effective irradiance experienced by each of the rods, melanopsin, and S, M, and L cones, using the light-measurement strategy proposed by a group of researchers^42^.

### Salivary Melatonin and Cortisol

Saliva samples were taken before (10:00) and after (11:00) the light exposure. Saliva samples were collected by participants spitting into a clear bottle from Salimetrics (PA, United States). The passive drool method was used because cotton swabs might interfere with the assay results of salivary melatonin^48^. Subjects were instructed to remain sitting on a chair during saliva collection because posture could affect hormone secretion as well^49^. Saliva samples were immediately frozen and kept below −20 °C until the melatonin and cortisol assays were conducted. Melatonin and cortisol levels of the samples were analysed in duplicate with commercially available enzyme-linked immunosorbent assay (ELISA; Salimetrics, PA, United States), and mean values of the duplicate were employed for elucidation. The minimum detectable dose of melatonin (analytical sensitivity) was determined to be 1.37 pg/ml, and the intra- and inter-assay coefficients of variation (CVs) were 7.4% and 22.8%, respectively. The assay sensitivity of cortisol was 0.007 μg/dL, and the intra- and inter-assay CVs were 7.5% and 15.1%, respectively.

### Subjective Perception of Sleepiness, Mood, and Visual Comfort

A set of subjective assessments was administered after the light exposure (11:00). Subjective sleepiness was rated using the KSS^50^, a nine-point scale ranging from 1 (extremely alert) to 9 (extremely sleepy, fighting sleep). Furthermore, participants were asked to assess their subjective mood and visual comfort on 100 mm VAS^43^, with the left end labelled “worst ever” and the right end labelled “best ever.” The participants were asked to mark on the 100 mm horizontal line the point that they felt represented their perception of their current state. Each score was expressed as a number between 0 (worst ever) and 100 (best ever).

### Statistical Analyses

All data were analysed using SPSS® version 20. Data were tested for normality using the Shapiro-Wilk test. A paired samples t-test was used for comparisons of percentage of melatonin change and a set of subjective assessments between the two light conditions. The Wilcoxon signed-rank test was used to compare the percentage of cortisol change between the two light conditions, as well as changes in salivary melatonin and cortisol concentrations before and after light exposure because not all data reached the criterion for normal distribution. In four saliva samples, we were unable to measure melatonin levels. The melatonin assay was performed after the cortisol assay, and in the case of two samples, there was not enough saliva for the melatonin assay. The other two cases were for saliva collected at 11:00, and one plausible reason might be that melatonin offset could have occurred earlier. All P values and confidence limits were based on two-tailed calculations. P values < 0.05 were considered significant.

## Electronic supplementary material

Dataset 1

## Data Availability

All data generated or analysed during this study are included in this published article (and its Supplementary Information files).
