# Supplementary material for: Awakening effects of blue-enriched morning light exposure on university students’ physiological and subjective responses
Source: Sci Rep. 2019 Jan 23;9:345. doi: 10.1038/s41598-018-36791-5 (PMC6344573; doi:10.1038/s41598-018-36791-5)
Supplement: Supplementary file 1 — Dataset 1 [file 41598_2018_36791_MOESM1_ESM.docx]

**Awakening effects of blue-enriched morning light exposure on university students’ physiological and subjective responses**

**Kyungah Choi^1^, Cheong Shin^2^, Taesu Kim^1^, Hyun Jung Chung^2^, and Hyeon-Jeong Suk^1^**

^1^Department of Industrial Design, Korea Advanced Institute of Science and Technology (KAIST), Daejeon 34141, Republic of Korea. ^2^Graduate School of Nanoscience and Technology, Korea Advanced Institute of Science and Technology (KAIST), Daejeon 34141, Republic of Korea. Correspondence and request for materials should be addressed to H.-J.S. (email: color@kaist.ac.kr)

**Supplementary Data**

Following the morning study, the evening study was conducted in succession. The specifics of the experimental setting were consistent with those of the previous study. Each session lasted for two hours. Saliva samples were taken before (22:00) and after (23:00) the light exposure. Unfortunately, the melatonin assays resulted in high assay failure rate of the evening data, especially for the pre-light exposure data. One plausible reason might be that the dim light melatonin onset (DLMO) could have occurred beyond these time intervals. Several reports place the DLMO between 19:30 and 23:00^1-3^. Consequently, data from the evening experiment were only reported here as supplementary information. In the evening, WL and BL exposure resulted in an increase of 96.90% and 34.76%, respectively, in comparison to pre-light exposure. Although complete paired comparison data from just five subjects were reported, the decline of melatonin levels was significantly greater after the exposure to BL (*t*(4) = 2.88, *p* = 0.045, Cohen’s *d* = 1.29). Comparison of salivary cortisol levels indicated that salivary cortisol concentration decreased 8.75% and 26.42% after WL and BL exposure, respectively. However, such a decrease in cortisol levels did not significantly differ between light conditions (*t*(14) = 1.77, *p* = 0.099, Cohen’s *d* = 0.46). These findings are, in general, in line with the data from the morning study reporting the effectiveness of blue-enriched morning light exposure on the salivary level of melatonin, but not on cortisol.

**Supplementary Table S1**. Results of the evening data. An asterisk indicates significance at *p* < 0.05. The means (s.e.m.) of experimental results are shown.

| **Measure** | **WL** | **BL** |
| --- | --- | --- |
| Melatonin change (%)* | 96.90 (48.79) | 34.76 (37.95) |
| Cortisol change (%) | –8.75 (9.58) | –26.42 (10.17) |

**Supplementary Reference**

1. Papaioannou, I. *et al*. Melatonin concentration as a marker of the circadian phase in patients with obstructive sleep apnoea. *Sleep Med.* **13,** 167-171 (2012).
2. Berry, R. B. & Wagner, M. H. *Sleep Medicine Pearls*. 3rd edn, 628 (Elsevier Health Sciences, 2014).
3. Hofstra, W. A. *et al*. Timing of temporal and frontal seizures in relation to the circadian phase: a prospective pilot study. *Epilepsy Res.* **94,** 158-162 (2011)
